# Improving the performance of supervised deep learning for regulatory genomics using phylogenetic augmentation

**DOI:** 10.1093/bioinformatics/btae190

**Published:** 2024-04-08

**Authors:** Andrew G Duncan, Jennifer A Mitchell, Alan M Moses

**Affiliations:** Cell & Systems Biology, University of Toronto, Toronto, ON M5S 3G5, Canada; Cell & Systems Biology, University of Toronto, Toronto, ON M5S 3G5, Canada; Cell & Systems Biology, University of Toronto, Toronto, ON M5S 3G5, Canada

## Abstract

**Motivation:**

Supervised deep learning is used to model the complex relationship between genomic sequence and regulatory function. Understanding how these models make predictions can provide biological insight into regulatory functions. Given the complexity of the sequence to regulatory function mapping (the cis-regulatory code), it has been suggested that the genome contains insufficient sequence variation to train models with suitable complexity. Data augmentation is a widely used approach to increase the data variation available for model training, however current data augmentation methods for genomic sequence data are limited.

**Results:**

Inspired by the success of comparative genomics, we show that augmenting genomic sequences with evolutionarily related sequences from other species, which we term phylogenetic augmentation, improves the performance of deep learning models trained on regulatory genomic sequences to predict high-throughput functional assay measurements. Additionally, we show that phylogenetic augmentation can rescue model performance when the training set is down-sampled and permits deep learning on a real-world small dataset, demonstrating that this approach improves data efficiency. Overall, this data augmentation method represents a solution for improving model performance that is applicable to many supervised deep-learning problems in genomics.

**Availability and implementation:**

The open-source GitHub repository agduncan94/phylogenetic_augmentation_paper includes the code for rerunning the analyses here and recreating the figures.

## 1 Introduction

Supervised deep learning approaches can predict chromatin accessibility (Kelley *et al.* 2016, Minnoye *et al.* 2020, Avsec *et al.* 2021a), transcription factor binding (Avsec *et al.* 2021b), enhancer activity (de Almeida *et al.* 2022), and other assays (Koo and Ploenzke 2020) from genomic sequence. The performance of trained deep learning models is measured using held-out test datasets, ruling out simple overfitting to limited training datasets. Aside from demonstrating powerful predictive performance, models that learn general biological features from experimental data can lead to an improved understanding of biological phenomena. Indeed, the application of various *post hoc* techniques (Shrikumar *et al.* 2020, Koo *et al.* 2021) has revealed that deep learning models trained on regulatory sequences learn both known and novel motifs and motif interactions (Kelley *et al.* 2016, Maslova *et al.* 2020, Minnoye *et al.* 2020, Avsec *et al.* 2021a,b, de Almeida *et al.* 2022, Novakovsky *et al.* 2023).

Using the tools of the deep learning era (Abadi *et al.* 2016, Paszke *et al.* 2019), computational biologists can now fit models with complexity that approaches the true biochemical complexity of transcriptional regulation (De Boer *et al.* 2020, Tareen and Kinney 2020). However, training models with 10^6^–10^8^ parameters require large and diverse datasets, which are available only for well-studied model organisms and human cell lines. A major challenge in the field is determining how to train more complex deep learning models for applications outside of the most data-rich systems. A proposed solution is to substantially increase data volume by performing assays on randomly generated synthetic sequences, and then evaluating models trained on these sequences using true genomic sequences (De Boer *et al.* 2020, de Boer and Taipale 2024). The reasoning behind this approach is that the genome does not contain sufficient variation to learn all aspects of the cis-regulatory code. However, this approach may not be suitable for all assays and requires additional experimentation.

Data augmentation is a technique used in deep learning to increase the performance of complex models by training models on transformed versions of the input data, thereby increasing the number of training examples. This technique finds application across a variety of fields including computer vision (Shorten and Khoshgoftaar 2019) and natural language processing (Li *et al.* 2022). However, there are limited domain-specific augmentation techniques for genomic sequence data. Two commonly used augmentation approaches include reverse complements (Cao and Zhang 2019) and genomic shifts (de Almeida *et al.* 2022, Toneyan *et al.* 2022). Recently, sequence augmentations inspired by evolution, including point mutations, inversions, and deletions, have shown promise in improving supervised model performance and interpretability (Lee *et al.* 2023). However, because these approaches introduce random sequence modifications to simulate evolution, they do not account for any functional constraint on the sequence.

Homologous sequences (or homologs for short) are genomic sequences from different species that share a common ancestor and may be under functional constraint but have likely diverged in primary sequence (Weirauch and Hughes 2010, Zoonomia Consortium 2020). Previously, homologs have been proposed as a form of sequence augmentation for self-supervised learning, as they can be thought of as transformed versions of their ancestral sequence that retain the same biological function (analogous to augmentations that do not alter the semantic meaning in images) (Kelley 2020, Lu *et al.* 2020, 2022). Additionally, it has been demonstrated that training deep learning models on functional genomic assays from both humans and mice can improve model performance compared to using data from a single species (Kelley 2020), indicating there is a benefit to training models on sequences from multiple species. Here, we investigate the use of homologs as a data augmentation method for supervised deep-learning models that predict functional genomic assays from genomic sequences. We find that augmenting genomic sequences with homologs can improve the performance of supervised deep-learning models. Furthermore, we show that phylogenetic augmentation can restore performance on models trained on down-sampled training sets, indicating that this approach has the potential to enhance data efficiency when training size is low. We then apply phylogenetic augmentation to a real-world small genomic dataset, where it allows for the training of a deep learning model.

## 2 Materials and methods

### 2.1 Datasets

We used published data from a massively parallel reporter assay (MPRA) experiment measuring enhancer activity across the *Drosophila melanogaster* genome in the S2 cell-line (de Almeida *et al.* 2022). The MPRA was performed once using a housekeeping promoter and once with a developmental promoter to investigate enhancer-promoter dependencies, resulting in two measurements per sequence. The dataset consists of 7062 and 11 658 enhancers identified in the housekeeping and developmental experiments, respectively. Additionally, 223 306 regions with varying activity levels were also included to provide sequences with a range of activity levels. All sequences are of length 249 bp. To match the original manuscript, chromosome 2R was split in half, with the first half used for validation and the second half used for testing. The remainder of the data was used for model training. Instead of including reverse complements in the dataset, reverse complements were randomly applied during model training to 50% of sequences.

The Basset dataset contains 2 071 886 DNase-seq peaks from 164 different human cell lines, along with a binary class for whether each peak is open in each cell type (Kelley *et al.* 2016). After filtering out sequences with Ns (unknown nucleotides), this was reduced to 2 021 532 regions. All sequences are of length 600 bp. To match the original manuscript, the data was split into training, validation, and testing using 93%, 3.5%, and 3.5% of the data, respectively.

The yeast 3′UTR dataset contains binary RNA-binding data for the PUF3 RNA-binding protein to 4293 3′UTRs from *Saccharomyces cerevisiae* (Gerber *et al.* 2004) that we obtained for a previous analysis (Alam *et al.* 2023). We selected PUF3 because in a previous analysis, we found that PUF3 had the strongest motif signal of the 74 RNA-binding proteins from AtTract (Giudice *et al.* 2016) in *S.cerevisiae* 3′UTRs (Alam *et al.* 2023). These 3′UTR sequences were defined as regions 200 bp downstream of the stop codon of a gene. Due to the limited number of total positives (198) in the dataset, we did not use a validation set to optimize parameters. Chromosome 4 (ChrYD) was used for the testing dataset, and the remaining chromosomes were used for model training.

### 2.2 Models

For the *Drosophila* S2 analysis, we used three different CNN architectures designed for DNA sequences and implemented them in TensorFlow: DeepSTARR(de Almeida *et al.* 2022), ExplaiNN (Novakovsky *et al.* 2023), and a custom architecture we call Motif DeepSTARR. These architectures are meant to represent various design choices used for deep learning of genomic sequences. The input to these models is 249-bp DNA sequences that are one-hot encoded, and the task is a regression on the MPRA enhancer activity for each promoter MPRA experiment. A summary of the encoder component of the architectures is provided below. The full model architectures are shown in Supplementary Fig. S4.

- **DeepSTARR**: A CNN composed of four convolutional layers followed by two fully connected layers. The convolutional layers are meant to capture sequence motifs, and the fully connected layers are meant to capture non-linear relationships between motifs.
- **Motif DeepSTARR**: A single convolutional layer with 256 filters, followed by two fully connected layers. The convolutional layer has a large filter size (19) in order to learn full motifs. The fully connected layers capture non-linear relationships between motifs.
- **ExplaiNN**: A linear combination of 256 motif representations. The motif representations are defined as a convolutional layer with one filter, followed by two fully connected layers.

For the Basset analysis, we used only the Basset model (Kelley *et al.* 2016). The input to this model is 600 bp DNA sequences that is one-hot encoded, and the task is 164 multi-label binary classification on 164 different human cell-lines. A summary of the encoder component of the architecture is provided below. The full model architecture is shown in Supplementary Fig. S5.

- **Basset**: A CNN composed of three convolutional layers followed by two fully connected layers. The inspiration for the DeepSTARR architecture.

For the yeast 3′UTR analysis, we used the same DeepSTARR encoder that was used with the *Drosophila* S2 analysis, however the input to the model was changed to 200-bp one-hot encoded DNA sequences. The task is binary classification of PUF3 binding, with 1 representing binding and 0 representing no binding.

### 2.3 Identifying homologous sequences from a multi-species genome alignment

For the *Drosophila* S2 analysis, we obtained an unpublished multi-species genome alignment of 168 genomes containing 137 species from the *Drosophila* genus, including *D.melanogaster* (Supplementary Data File S1). Homologs from other *Drosophila* species were extracted from the alignment for each *D.melanogaster* training sequence using HAL (Hickey *et al.* 2013) and the HALPER tool (Zhang *et al.* 2020). If multiple homologs were found in a target organism, the first match was arbitrarily selected. The selected sequences were then resized to 249 bp from the midpoint to match the length of the training sequence. This resulted in 10 582 901 homologs in total across all 191 109 sequences in the training set.

For the Basset analysis, we used the Zoonomia multi-species genome alignment of 241 mammalian species (Zoonomia Consortium 2020). Homologs from 42 species were used for this analysis and were restricted to clades around the *Homo sapiens* node (Supplementary Data File S2). The selected sequences were then resized to 600 bp from the midpoint to match the length of the training sequence. This resulted in 62 538 898 homologs in total across all 1 880 029 sequences in the training set.

For the yeast 3′UTR analysis, we used a previously collected dataset of homologs from Ensembl (Zerbino *et al.* 2017) for 24 different yeast species (Supplementary Data File S3) (Alam *et al.* 2023). Unlike the multi-species genome alignment approach that we had used to identify homologs for the *D.melanogaster* and *H.sapiens* sequences in the previous analyses, we used a genic approach to identify the *S.cerevisiae* homologs. Briefly, the Ensembl REST API (Yates *et al.* 2015) was used to retrieve one-to-one orthologs for each gene along with the 200 bp downstream of the stop codon of the corresponding gene. This resulted in a total of 71 498 homologs across all 3721 sequences in the training set.

### 2.4 Model training and fine-tuning

For the *Drosophila* analysis, models were trained using TensorFlow (Abadi *et al.* 2016) with the Adam optimizer (Kingma and Ba 2017) for 100 epochs with a learning rate of 2 × 10^-3^, mean squared error loss and early stopping of 10. During model training, phylogenetic augmentation was applied to each training sequence (phylogenetic augmentation rate = 1.0 unless otherwise specified). This was done by sampling from the set of homologs for a given training sequence. A reverse complement augmentation was also applied randomly to sequences, including when no phylogenetic augmentation was used. As in a recent study of sequence augmentations, after training the models using the phylogenetic augmentation approach, we fine-tuned for five epochs at a lower learning rate (1 × 10^-4^) using only the original training sequences (Lee *et al.* 2023). Phylogenetic augmentation is never applied to the validation or testing sets. For the sampling experiment, we trained the DeepSTARR model with sampled fractions of the training data. Sampling was performed separately for each replicate. We use the Pearson correlation coefficient (PCC) to measure each model’s performance for each task on the same held-out test dataset.

The Basset models were trained similarly to the *Drosophila* analysis, with the following changes. Models were trained with the Adam optimizer for 20 epochs with a learning rate of 2 × 10^-3^ and binary cross entropy loss. The average area under the precision–recall curve (avg AUPRC) was used to measure the model’s performance for each task on the held-out test dataset. This was used in place of the average area under the curve (AUC), as the data has a large class imbalance between positives and negatives.

The yeast models were trained similarly to the *Drosophila* analysis, with the following changes. Models were trained with the Adam optimizer for 50 epochs. No early stopping on the validation set was done, as there was no validation set. The AUPRC)was used to measure the model’s performance for the classification task on the held-out test dataset. Due to the small size of the dataset, six replicates were trained for each model. Additionally, as RNA is single-stranded, reverse complements were not applied. As a negative control, the model was trained on the training data with the labels scrambled.

All models were trained using an NVIDIA RTX A400 GPU with 16376MiB of memory.

### 2.5 Global importance analysis of PUF3 motif

A set of 1000 sequences of length 200 bp were randomly generated to use as a background set for the analysis. The PUF3 consensus motif (TGTAAATA) (Hogan *et al.* 2015) was randomly inserted once into each background sequence to create a set of sequences with the PUF3 motif. As a control, we also created a set of sequences with a scrambled PUF3 consensus motif randomly inserted. A replicate of the baseline model and a replicate of the phylogenetic augmentation with fine-tuning model were used to predict the class of each sequence.

### 2.6 Investigating hyperparameters

For the number of species analysis, the *Drosophila* phylogenetic tree was extracted from the *Drosophila* multi-species Cactus alignment (Paten *et al.* 2011) using the halStats command (–tree) from the HAL package (Hickey *et al.* 2013). The ETE Toolkit (Huerta-Cepas *et al.* 2016) get_distance function was used to determine the distance between *D.melanogaster* and all other species in the phylogenetic tree. These species were then sorted by their ascending evolutionary distance. The DeepSTARR model was then trained using phylogenetic augmentation on the homologs for the closest species, then the two closest species, and so on. For each set of species, three DeepSTARR replicates were trained. To measure the total evolutionary distance from *D.melanogaster* for each set of species, the ETE Toolkit prune function (preserve_branch_length = True) was used to prune all but the current set of species from the species tree. The total distance was then calculated by summing all the branch lengths in the pruned Newick tree. The branch lengths are measured using substitutions per site of the 4-fold degenerate sites of BUSCO genes (Kim *et al.* 2023).

For the phylogenetic augmentation rate analysis, the DeepSTARR model was trained on the *Drosophila* S2 dataset using increasing rates of phylogenetic augmentation. Homologs for all 136 *Drosophila* species from the alignment were used. Three replicates were run for each rate of augmentation.

Training of Basset models took substantially more time due to the length of the sequences and the dataset being an order of magnitude larger than the DeepSTARR data. Therefore, hyperparameters were not explored in Basset. Additionally, we did not investigate hyperparameters for the yeast data due to the limited data size.

## 3 Results

### 3.1 Phylogenetic augmentation: a method for augmenting genomic sequences using multi-species genome alignments

We define phylogenetic augmentation for supervised deep learning as the transformation of a genomic sequence from one species into a homolog from another species. This approach leverages homologs extracted from multi-species genome alignments to improve the diversity of input training data. By presenting these homologs as augmented versions of training sequences, deep learning models see a broader array of sequences during the training process.

**Figure 1.**
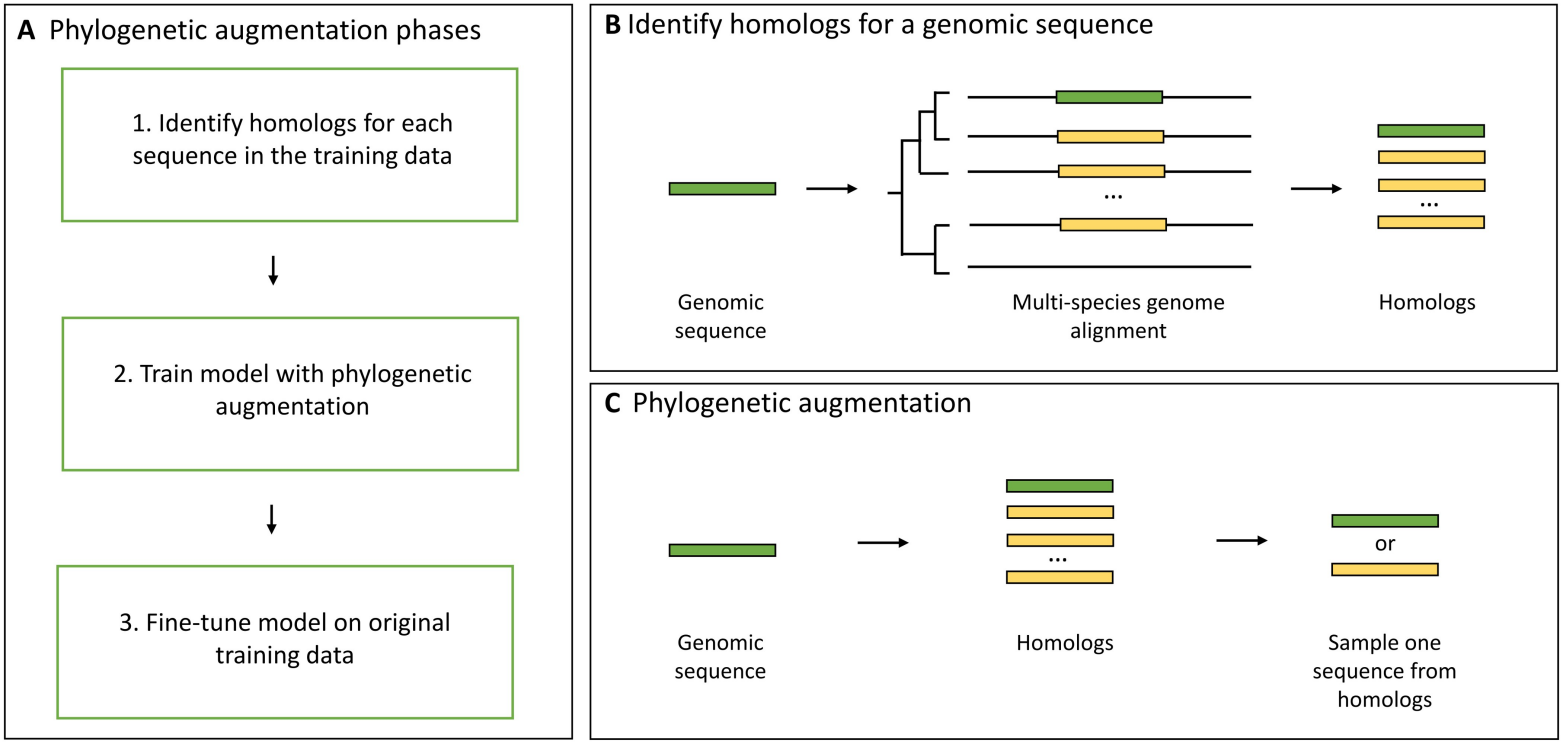
An overview of the phylogenetic augmentation method. (A) The three phases of phylogenetic augmentation for model training. (B) During preprocessing, homologs (yellow boxes) of each training sequence (green boxes) are identified in different genomes (black lines) using multi-species genome alignments. (C) Phylogenetic augmentation is implemented as the transformation of a genomic sequence to a random homologous sequence.

The application of phylogenetic augmentation within supervised deep learning problems involves three phases, as illustrated in Fig. 1A. Prior to model training, an initial preprocessing step is performed to extract homologs for each genomic sequence in the training set from a multi-species genome alignment that contains the species being investigated (Fig. 1B). This is done before training so that existing alignment tools can be used to extract homologs (see Section 2). During model training, phylogenetic augmentation is applied to all training sequences at batch generation (Fig. 1C). This significantly increases the number of training examples that the model encounters. Following a previous approach, after training, the models are fine-tuned on the original genomic sequences to further improve performance to reduce potential bias from including functionally diverged homologs (Lee *et al.* 2023).

### 3.2 Phylogenetic augmentation improves CNN prediction performance on held-out test sets

To investigate whether phylogenetic augmentation used on a supervised deep learning problem improved model performance, we trained convolutional neural networks (CNNs) with phylogenetic augmentation to predict *Drosophila* S2 STARR-seq activity driven by a housekeeping or developmental promoter (de Almeida *et al.* 2022), and compared performance to a baseline where no phylogenetic augmentation or fine-tuning was applied. For each sequence in the training set, homologs for 136 species in the *Drosophila* genus were extracted from a multi-species genome alignment. To explore the effect of CNN architecture, three CNN architectures of varying complexities were trained on this data [DeepSTARR (de Almeida *et al.* 2022), ExplaiNN (Novakovsky *et al.* 2023), and Motif DeepSTARR], and their performance was measured on a held-out test set (see Section 2).

**Figure 2.**
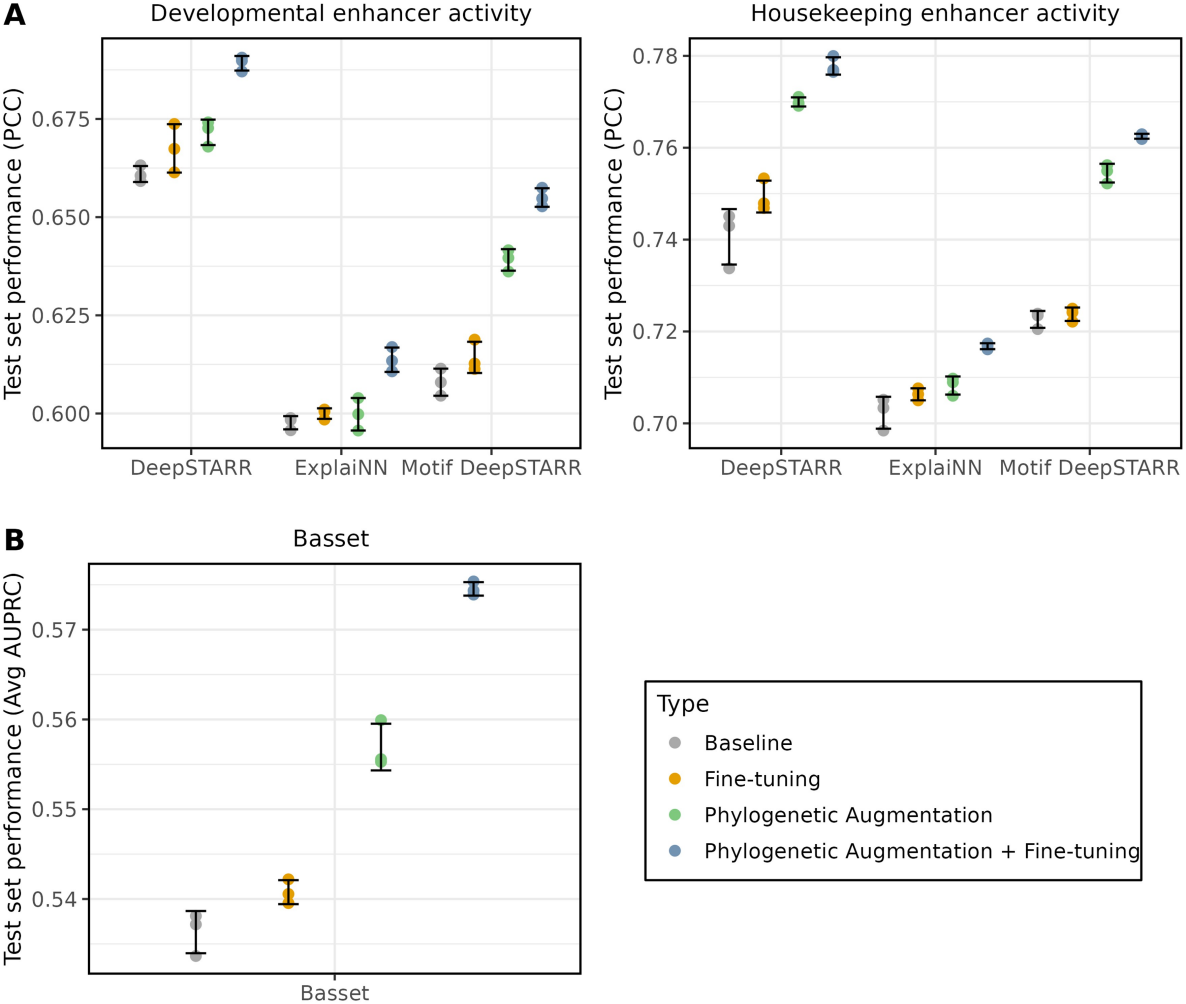
Phylogenetic augmentation improves model performance with various CNN architectures. (A) CNN test performance (PCC) is shown on the y-axis for the *Drosophila* S2 Developmental (left) and Housekeeping (right) enhancer activity for trained models. (B) Basset model test performance (avg AUPRC) is shown on the *y*-axis for 164 cell-type specific chromatin accessibility (DNase-seq) experiments for trained models. (A and B) The grey points are the baseline models, which were trained with no phylogenetic augmentation or fine-tuning. The yellow points are the baseline models that have been fine-tuned on the original data. The green points are models trained with phylogenetic augmentation. The blue points are the models trained with phylogenetic augmentation that have been fine-tuned on the original data. The black error bars represent the standard deviation of the three replicates for each CNN architecture and dataset.

We observed that all CNN models demonstrated an increase in test set performance compared to the baseline with the inclusion of phylogenetic augmentation and fine-tuning (Fig. 2A). For example, the average DeepSTARR model performance (PCC) increased from 0.661 (±<0.01 SE) to 0.689 (±<0.01 SE) (+4.2%) for the developmental enhancer activity, and 0.741 (±<0.01 SE) to 0.779 (±<0.01 SE) (+5.1%) on the housekeeping enhancer activity (grey and blue points). The ExplaiNN CNN had a smaller performance increase than the other two architectures. This can be attributed to differences between the ExplaiNN and the DeepSTARR models. ExplaiNN uses linear combinations of learned motif representations to make predictions, and unlike the other two CNN models which have fully connected layers, cannot learn complex non-linear interactions between different transcription factor motifs (Novakovsky *et al.* 2023). While fine-tuning the models on the original training data after phylogenetic augmentation further improved performance (green points and blue points), fine-tuning the baseline models was not sufficient by itself to achieve the same level of performance increase seen with phylogenetic augmentation and fine-tuning (yellow points and blue points).

Next, we performed a similar analysis using the Basset model to predict binary DNase-seq peaks across 164 human cell lines, which we refer to as the Basset dataset (Kelley *et al.* 2016). For every sequence in the training set, homologs from a clade of 43 species that included *Homo sapiens* were extracted from a mammalian genome alignment. Again, we observed improved model performance on a held-out test set when comparing the baseline models to the models with phylogenetic augmentation and fine-tuning, with the AUPRC increasing from 0.536 (±<0.01 SE) to 0.575 (±<0.01 SE) (+7.2%) (Fig. 2B). Together, these results demonstrate that phylogenetic augmentation is a useful data augmentation approach for training supervised deep learning models on genomic sequences, though the magnitude of improvement is dependent on model architecture.

### 3.3 Phylogenetic augmentation improves data efficiency in supervised deep learning

**Figure 3.**
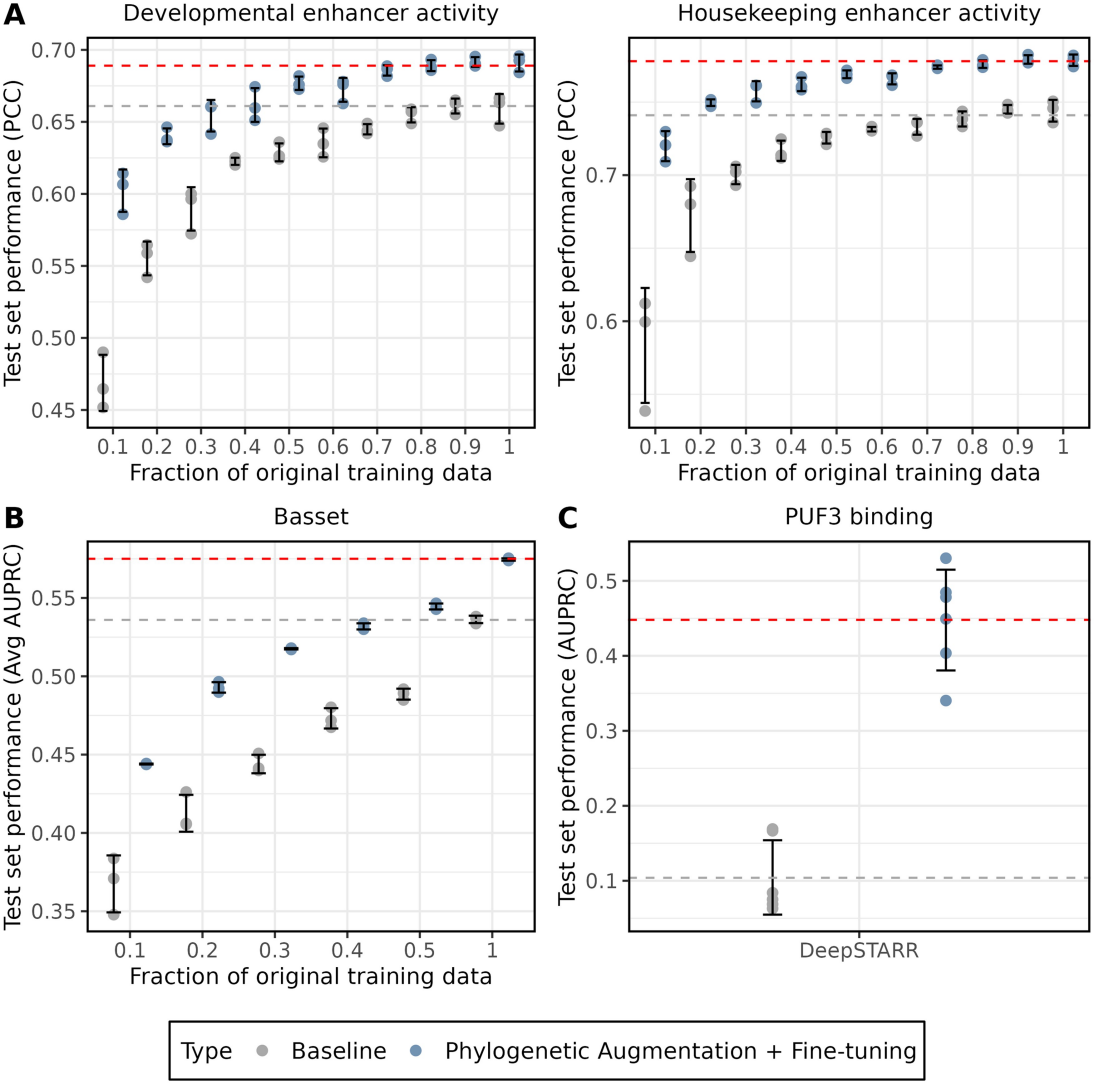
Phylogenetic augmentation improves data efficiency when training size is reduced. (A) DeepSTARR test performance (PCC) is shown on the *y*-axis for the *Drosophila* S2 Developmental (left) and Housekeeping (right) enhancer activity for trained models. (B) Basset test performance (avg AUPRC) is shown on the *y*-axis for 164 cell-type specific chromatin accessibility (DNase-seq) experiments for trained models. (A, B) The *x*-axis represents the fraction of the original training data that was sampled during model training. (C) The DeepSTARR test performance (AUPRC) is shown on the *y*-axis for the binary classification prediction of *S.cerevisiae* 3′UTR PUF3 binding. (A–C) The grey points are the baseline models, which were trained with no phylogenetic augmentation or fine-tuning. The blue points are the models trained with phylogenetic augmentation that have been fine-tuned on the original training data. The black error bars represent the standard deviation of the replicates for each CNN architecture and dataset. The dotted grey line represents the average test performance on the original training data with no phylogenetic augmentation or fine-tuning. The dotted red line represents the average test performance on the original training data with phylogenetic augmentation and fine-tuning.

Some regulatory datasets may not include enough regions of interest for effective machine learning, which could lead to overfitting on training data. Phylogenetic augmentation could serve as a method for improving data efficiency when training models on smaller genomic datasets. To investigate this, we down-sampled different fractions of the training sequences for the *Drosophila* S2 STARR-seq dataset and applied phylogenetic augmentation during DeepSTARR model training to determine if test set performance could be rescued. For each fraction of the training sequences, phylogenetic augmentation with fine-tuning improved test set performance compared to the baseline (blue and grey points) (Fig. 3A). At 40% and 20% of the original training sequences, phylogenetic augmentation plus fine-tuning was sufficient to rescue the baseline model’s performance on the test set for the developmental and housekeeping enhancer activities, respectively (Fig. 3A, dotted grey lines). The largest performance improvements were seen when phylogenetic augmentation and fine-tuning were applied to only 10% of the dataset, with a gain over the baseline model of 0.133 for the developmental enhancer activity compared to an improvement of 0.0318 on 100% of the original dataset (Fig. 3A). A similar experiment was performed for the Basset dataset using the Basset model, with phylogenetic augmentation plus fine-tuning again improving test-set performance for all fractions tested (Fig. 3B). At around 40% of the original training data, phylogenetic augmentation was able to rescue the baseline model performance seen with all the training data (Fig. 3B, dotted grey line). These results indicate that phylogenetic augmentation can enhance the data efficiency of supervised deep-learning models, enabling them to make better predictions with less data.

Due to the large performance increases seen on the down-sampled *Drosophila* dataset, we next asked whether phylogenetic augmentation could be applied to a real-world example of a small dataset where it is challenging to train a complex deep learning model. We chose to predict the binding of the RNA-binding protein PUF3 to 3′ untranslated regions (3′UTRs) of *S.cerevisiae* (Alam *et al.* 2023) because this dataset is more than an order of magnitude smaller than the *Drosophila* dataset (∼5000 *S.cerevisiae* 3′UTRs versus ∼200 000 *Drosophila melanogaster* STARR-seq regions). The hyperparameters controlling the complexity of the DeepSTARR model were optimized for the *D.melanogaster* S2 STARR-seq dataset, and the model contains 500 000 parameters. We reasoned that a model of this complexity would be challenging to train on the *S.cerevisiae* 3′UTRs.

To test this, we trained a DeepSTARR model as above, and as expected, found that the DeepSTARR model had a test classification performance (AUPRC) of 0.104 (±<0.1 SE) on the test data (Fig. 3C), only marginally better than the test performance of 0.0427 (±<0.01 SE) seen on a scrambled label control. Remarkably, when phylogenetic augmentation and fine-tuning were applied to this dataset during model training, the average test performance increased by over 4-fold to 0.448 (±<0.1 SE) (Fig. 3C). To test the biological relevance of this performance increase, we wondered whether the large test performance increase could be explained by the augmented model learning the known PUF3 consensus motif (Hogan *et al.* 2015). We performed a global importance analysis (Koo *et al.* 2021) of the PUF3 motif for both the baseline model and the model with phylogenetic augmentation and fine-tuning (see Section 2). Only with phylogenetic augmentation does the model place importance on the PUF3 motif (Supplementary Fig. S1). Taken together, these results demonstrate that phylogenetic augmentation can enable the training of complex deep learning models and learning of biologically relevant features on small genomic datasets.

### 3.4 Exploring hyperparameters for phylogenetic augmentation

To assess the impact of different hyperparameters on phylogenetic augmentation, we trained multiple DeepSTARR models on the *Drosophila* S2 STARR-seq dataset and varied the number of species used and the rate at which phylogenetic augmentation was applied.

**Figure 4.**
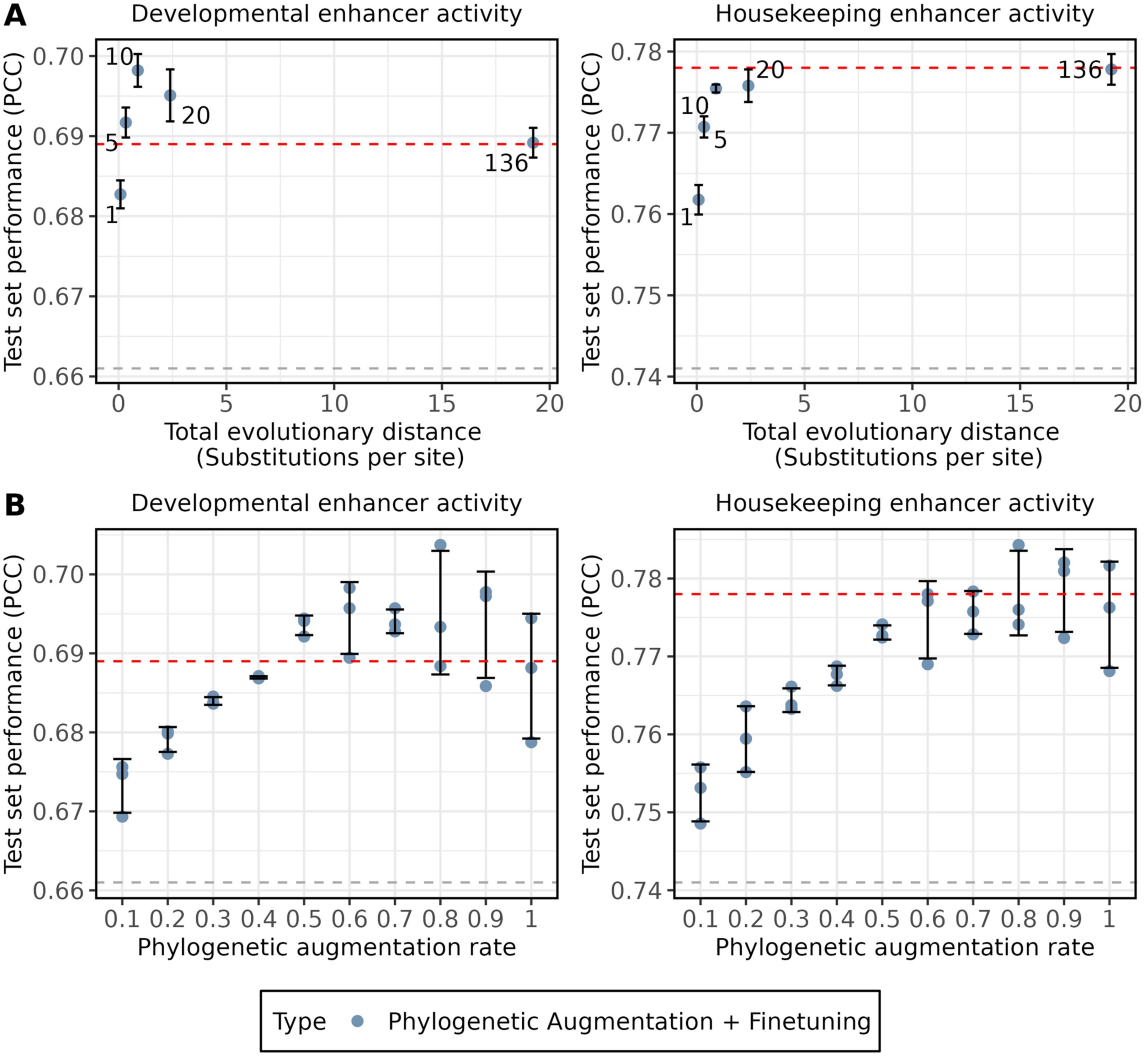
Hyperparameters for phylogenetic augmentation impact test set performance. (A) DeepSTARR test performance (PCC) is shown on the *y*-axis for Developmental (left) and Housekeeping (right) enhancer activity for trained models. The *x*-axis represents the total evolutionary distance of species included during phylogenetic augmentation, measured using substitutions per site. The labels represent the total number of species used to train each model. The blue dots represent the average test set performance across replicates. (B) DeepSTARR test performance (PCC) is shown on the *y*-axis for Developmental (left) and Housekeeping (right) enhancer activity for trained models. The *x*-axis represents the rate at which phylogenetic augmentation is applied during model training. The blue dots represent the test set performance for individual replicates. (A and B) The black error bars represent the standard deviation of the three replicates that were trained for each number of species. The dotted grey line represents the average test performance on the original training data with no phylogenetic augmentation or fine-tuning. The dotted red line represents the average test performance on the original training data with phylogenetic augmentation and fine-tuning using all 136 *Drosophila* species and a phylogenetic augmentation rate of 1.

First, we examined how the number of species and their total evolutionary distance used during phylogenetic augmentation impacted model test performance. We arranged the *Drosophila* species from the *Drosophila* multi-species alignment based on increasing evolutionary distance from *D.melanogaster*. Then, we trained multiple DeepSTARR models with phylogenetic augmentation and fine-tuning, progressively incorporating more distant species from which to draw homologs. We observed that including homologs for just one additional species yielded a noticeable performance increase for both enhancer activity measurements (Fig. 4A). Interestingly, model test performance improvements plateaued around 10 species for housekeeping enhancer activity, but for developmental enhancer activity performance started decreasing after 10 species. Performing the same analysis starting with the most distant species had minimal test set performance increases for the first 20 species, though there was some improvement over the baseline model (Supplementary Fig. S2). These results suggest that a handful of closely related species is sufficient for improving model test performance through phylogenetic augmentation and that including too many distant species may decrease phylogenetic augmentation performance gains in some cases.

Next, we explored how the rate at which phylogenetic augmentation is applied during training affects test performance. We trained DeepSTARR models on the *Drosophila* S2 STARR-seq dataset, applying different rates of phylogenetic augmentation at batch generation. For example, a rate of 0.5 signified that 50% of the sequences in each batch underwent phylogenetic augmentation. This was carried out using homologs for all 136 *Drosophila* species from the *Drosophila* multi-species alignment. As shown in Fig. 4B, an increase in the rate of phylogenetic augmentation led to improved test set performance for both enhancer activity measurements. However, past a rate of 0.5, test set performance became more variable and subsequently decreased or plateaued. We hypothesized that the performance variation was caused by the higher phylogenetic augmentation rates adding too many sequences with diverged functions. Consistent with this hypothesis, the effect was reduced when we repeated the analysis with the 10 closest species (Supplementary Fig. S3). These results indicate the importance of the rate of phylogenetic augmentation when using this augmentation approach, and that it is not always the best to apply this augmentation to every sequence in a batch.

## 4 Discussion

Here, we introduced a data augmentation method for supervised deep learning of genomic sequences that takes advantage of multi-species genome alignments and the phylogenetic relationship between homologous sequences. This approach improved the performance of regression and classification problems for three different functional genomic datasets across two different kingdoms of life, indicating it is a general approach for augmenting genomic sequence data. Another way to use homologs during training is by transferring the labels from reference sequences to homologous sequences (Mourad 2024), which is expected to give similar results as the augmentation approach considered here. Additionally, we used phylogenetic augmentation on a reduced dataset to rescue model performance seen with the original training dataset, demonstrating that this approach improves the data efficiency of models.

In machine learning, data augmentation finds common use when datasets are too small such that deep learning models will memorize or overfit the training data. Although many regulatory datasets, such as those resulting from functional genomic experiments, contain large quantities of data, not all do. For instance, curated databases like the VISTA enhancer database include only a few thousand mouse and human enhancers (Visel *et al.* 2007). Potentially, data augmentation approaches like phylogenetic augmentation could contribute to addressing the challenge of training deep learning models on these small genomic datasets. Our results suggest that phylogenetic augmentation is most effective on small datasets (Fig. 3A). In line with this observation, applying phylogenetic augmentation to a DeepSTARR model trained on PUF3 binding across *S.cerevisiae* 3′UTRs resulted in a substantial increase in test set performance over the baseline model (Fig. 3C) and the ability to learn the PUF3 consensus motif (Supplementary Fig. S1).

Regulatory elements, like promoters and enhancers, undergo evolution at different rates, which can lead to varying rates of element turnover (Villar *et al.* 2015). Thus, the efficacy of this approach can be influenced by the species chosen for augmentation, as some elements may lose function quicker than others. Since the *Drosophila* S2 STARR-seq data contains enhancers, which have higher rates of turnover, we restricted species to those in the *Drosophila* genus. Had we included only species outside of the genus, such as other insects, the number of homologs identified would have been noticeably lower. Consequently, the phylogenetic augmentation method likely would have had a decrease in effectiveness. We observed that even for the same type of regulatory elements, there can be differences in the effectiveness of distant species. Test performance increases from applying phylogenetic augmentation to the *Drosophila* S2 developmental enhancers peaked at 10 species before slowly falling (Fig. 4A). Meanwhile, the test performance of the housekeeping enhancers continued to increase (albeit minimally) with the number of species (Fig. 4A). A potential explanation is that the expression of housekeeping genes is more conserved than tissue-specific genes (She *et al.* 2009), suggesting housekeeping enhancers may be under stronger functional conservation. Based on these results, we recommend using 10 closely related species as a starting point when applying phylogenetic augmentation during model training.

While it has been demonstrated that training a CNN to predict both mouse and human functional genomic experiments from DNA sequence can improve predictive performance on an individual species (Kelley 2020), this approach requires data from multiple assays which require additional time, resources, and expertise. While there may be data from relevant experimental assays available, these are often restricted to a handful of well-studied species (e.g. ENCODE Project). Our approach does not have these limitations, as it only requires sequenced genomes and whole genome alignments. In the case where there are no available multi-species genome alignments that include a species of interest, evolutionary augmentation using simulated mutations can be applied (Lee *et al.* 2023), or alignments can be generated from resources such as the Earth Biogenome Project (Lewin *et al.* 2018) or the NCBI genome database (Sayers *et al.* 2022). We recommend creating custom Cactus alignments (Armstrong *et al.* 2020) using HAL (Hickey *et al.* 2013), which was designed to work with large multi-species genome alignments.

As researchers are increasingly relying on the features learned by deep learning models trained on sequence data to obtain biological insights into regulatory function, it is crucial that these models are not overfitting to their training data and generalize to unseen sequences. The method we proposed here holds the potential for application in any supervised deep learning problem that uses non-coding genomic sequences as input, even when the number of training examples is insufficient for complex deep learning models. With the increasing availability of large alignments such as the 240-way mammalian alignment from Zoonomia (Zoonomia Consortium 2020), the 239-way primate alignment from Zoonomia (Kuderna *et al.* 2023), the 341-way avian alignment from B10K (Feng *et al.* 2020), and the 168-way *Drosophila* alignment used here (Supplementary Table S5), our approach is widely applicable across a variety of species.

## Supplementary Material

btae190_Supplementary_Data

## Data Availability

The data underlying this article are available on Zenodo (10.5281/zenodo.10463493). The *Drosophila* multi-species genome alignment that we used for this manuscript is available upon request from Dr Bernard Kim (Stanford University). Additionally, a newer version of the *Drosophila* multi-species genome alignment is available that includes additional species (Kim *et al.* 2023) (Supplementary Table S5).
