# Supplementary material for: Improving the performance of supervised deep learning for regulatory genomics using phylogenetic augmentation
Source: Bioinformatics. 2024 Apr 8;40(4):btae190. doi: 10.1093/bioinformatics/btae190 (PMC11042905; doi:10.1093/bioinformatics/btae190)
Supplement: btae190_Supplementary_Data [file btae190_supplementary_data.zip › Phylo_Aug_Supplementary_Figures.pdf]

Supplementary figures

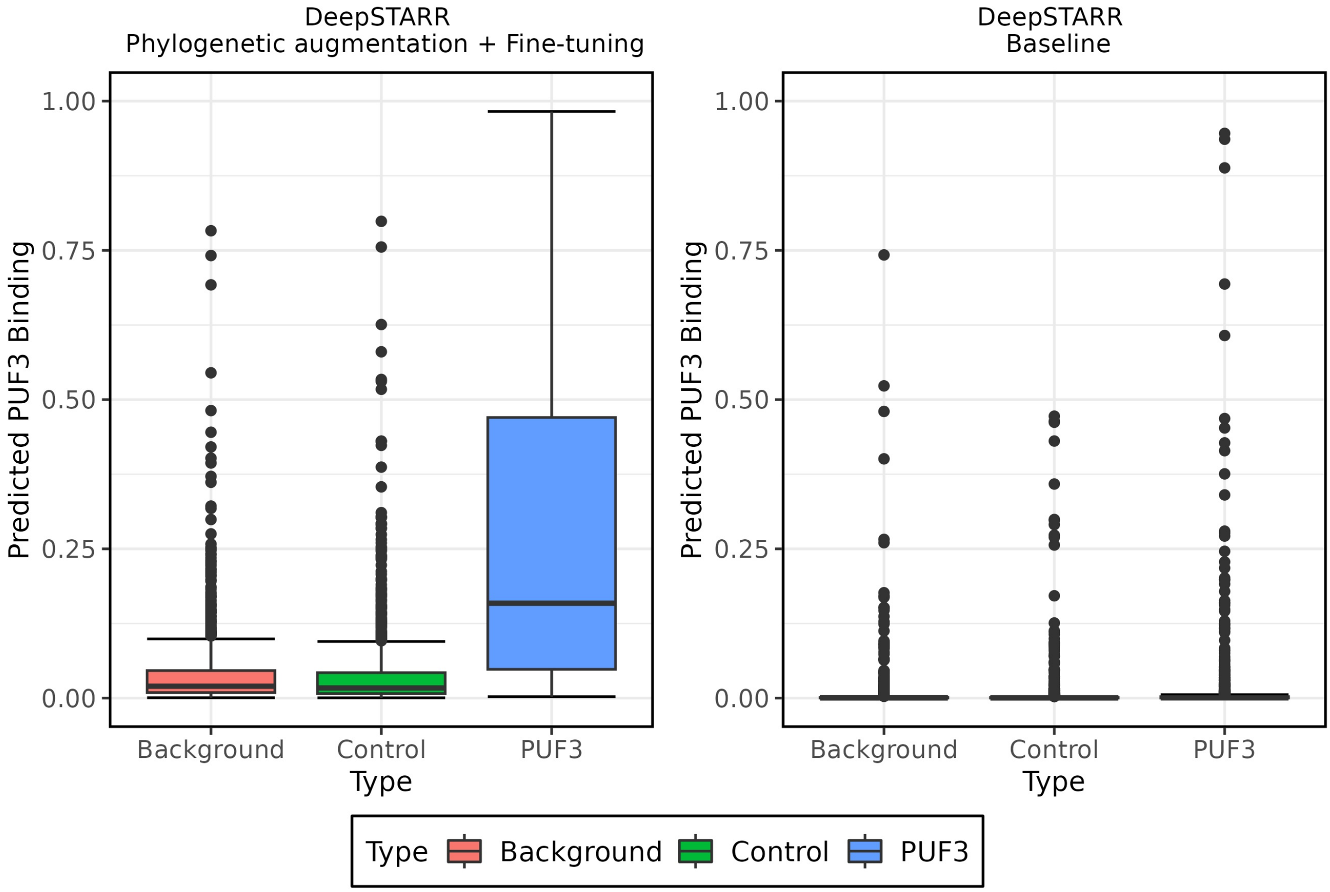

Supplementary Figure 1: **Global importance analysis of the PUF3 consensus motif in the DeepSTARR models.** Predicted PUF3 binding class probability for a DeepSTARR model trained with phylogenetic augmentation and fine-tuning (left) and for a baseline DeepSTARR model (right). The x-axis is the type of sequence set and the y-axis is the predicted PUF3 binding (between 0 and 1). The background type is 1,000 random sequences of length 200bp. The PUF3 type contains the 1,000 background sequences with the PUF3 consensus motif randomly inserted. The control type contains the 1,000 background sequences with a scrambled PUF3 consensus motif randomly inserted.

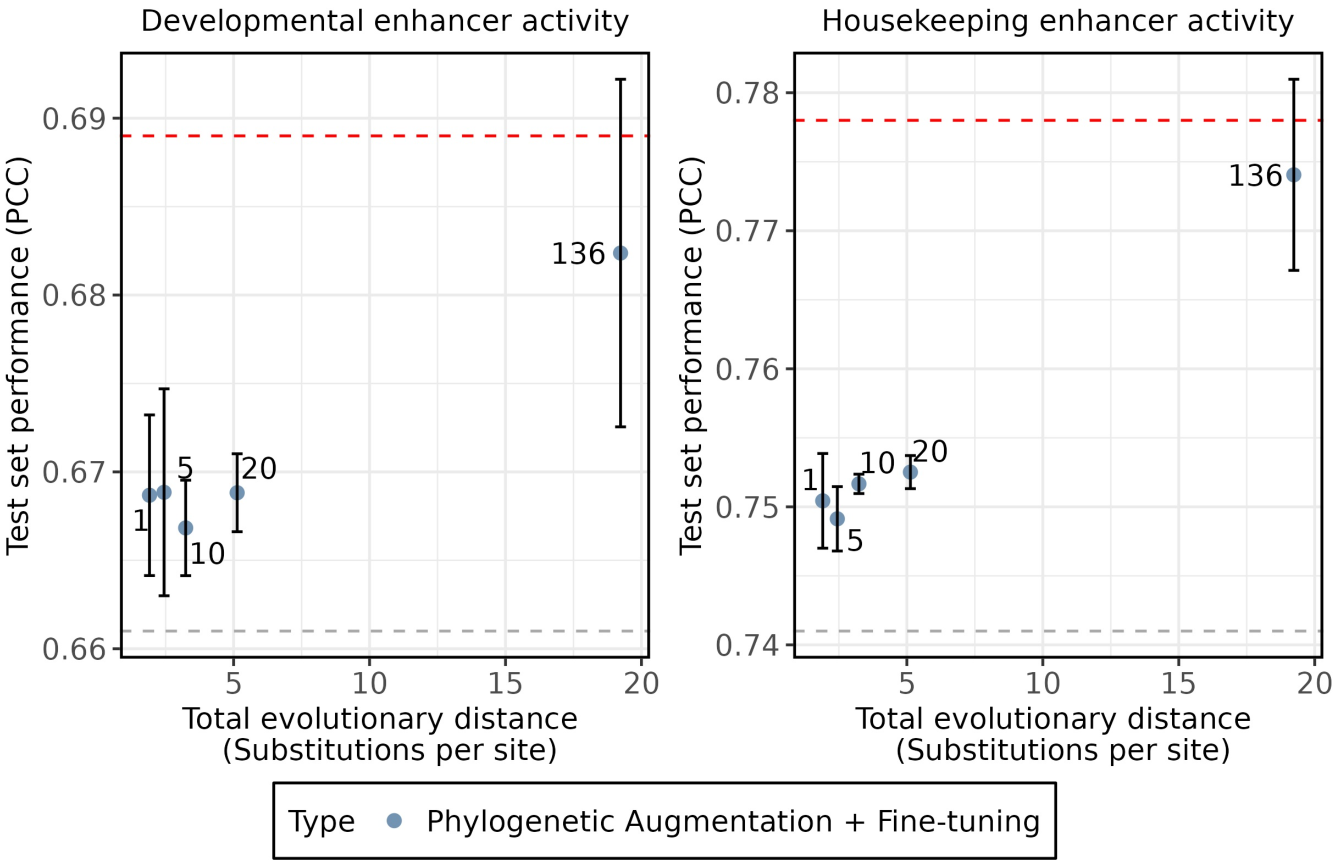

Supplementary Figure 2: **Hyperparameter analysis of species with decreasing evolutionary distance.** DeepSTARR test performance (Pearson correlation coefficient; PCC) is shown on the y-axis for *Drosophila* S2 Developmental (left) and Housekeeping (right) enhancer activity for trained models. The x-axis represents the total evolutionary distance of the species used during phylogenetic augmentation with *D. melanogaster*. The labels represent the total number of species used to train each model. The blue dots represent the average test set performance across replicates. The black error bars represent the standard deviation of the three replicates that were trained for each number of species. The dotted grey line represents the average test performance on the original training data with no phylogenetic augmentation or fine-tuning. The dotted red line represents the average test performance on the original training data with phylogenetic augmentation and fine-tuning using all 136 *Drosophila* species and a phylogenetic augmentation rate of 1.

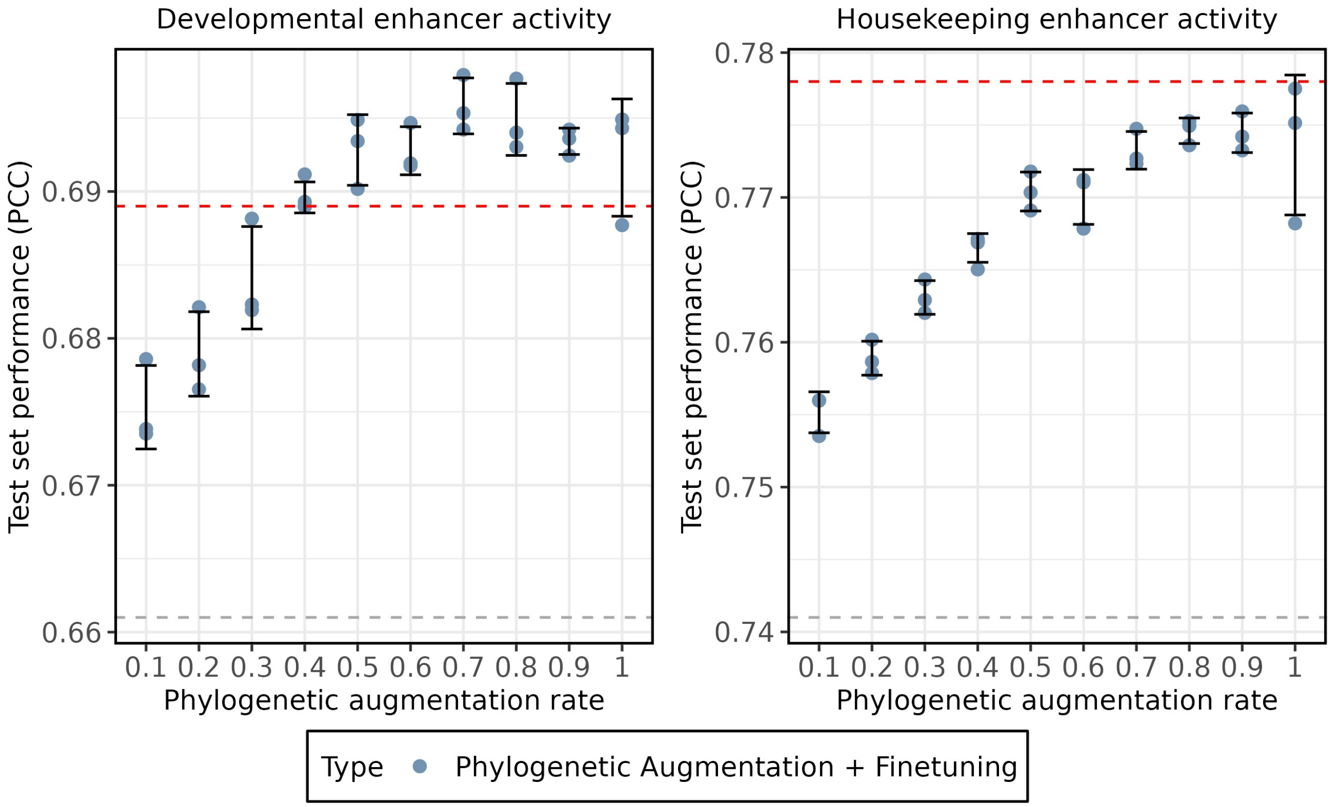

Supplementary Figure 3: **Hyperparameter analysis of phylogenetic augmentation rate with 10 closely related species.** DeepSTARR test performance (Pearson correlation coefficient; PCC) is shown on the y-axis for *Drosophila* S2 Developmental (left) and Housekeeping (right) enhancer activity for trained models. The x-axis represents the rate at which phylogenetic augmentation is applied during model training. The blue dots represent the test set performance for individual replicates. The black error bars represent the standard deviation of the three replicates that were trained for each number of species. The dotted grey line represents the average test performance on the original training data with no phylogenetic augmentation or fine-tuning. The dotted red line represents the average test performance on the original training data with phylogenetic augmentation and fine-tuning using all 136 *Drosophila* species and a phylogenetic augmentation rate of 1.

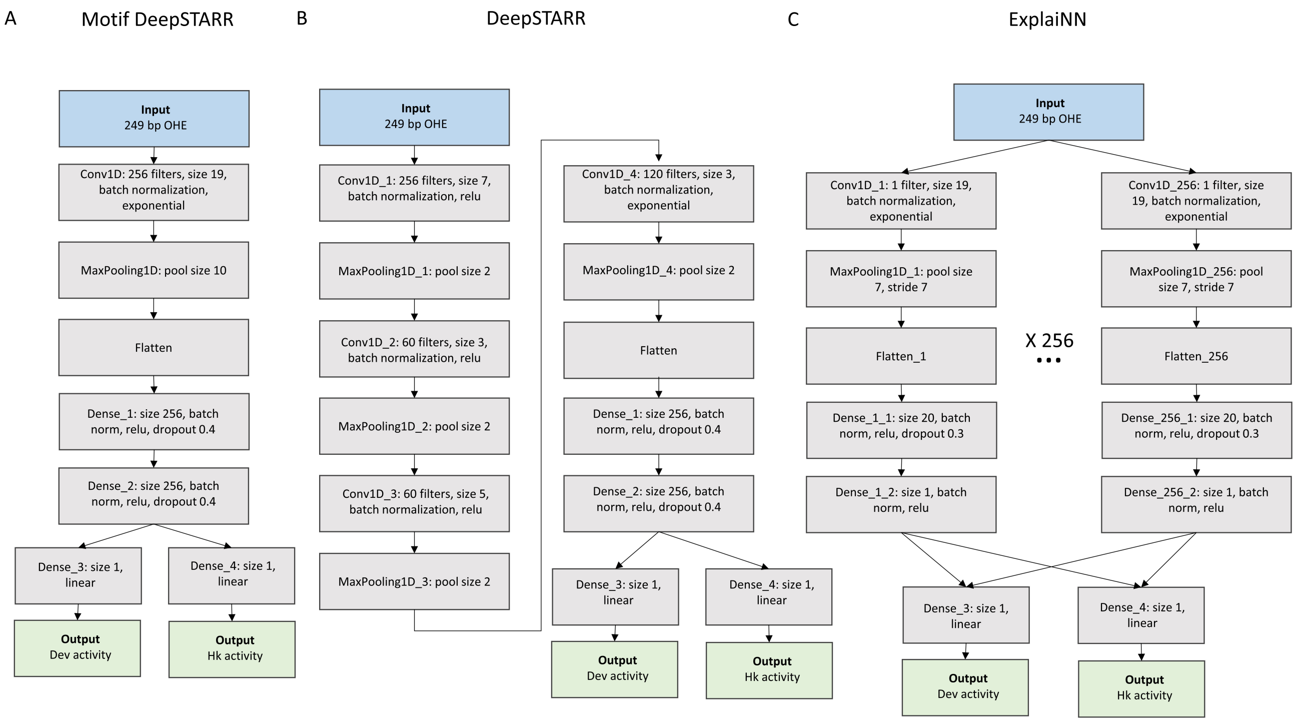

Supplementary Figure 4: **Architecture of the *Drosophila* deep learning models.** (A) The Motif DeepSTARR model is a modified version of the DeepSTARR model (de Almeida et al., 2022). The 4 convolutional layers were replaced with a single convolutional layer meant to learn full motifs. (B) The DeepSTARR model (de Almeida et al., 2022) was designed to predict developmental (dev) and housekeeping (hk) enhancer activity from one-hot encoded (OHE) 249bp DNA sequence for *Drosophila melanogaster* S2 STARR-seq data. (C) The ExplaiNN model (Novakovsky et al., 2023) was designed to use a linear combination of learned motifs to predict functional genomics data. (A-C) The grey boxes represent layers and contain layer name/type and parameters. The blue boxes represent the input and the green boxes represent the output.

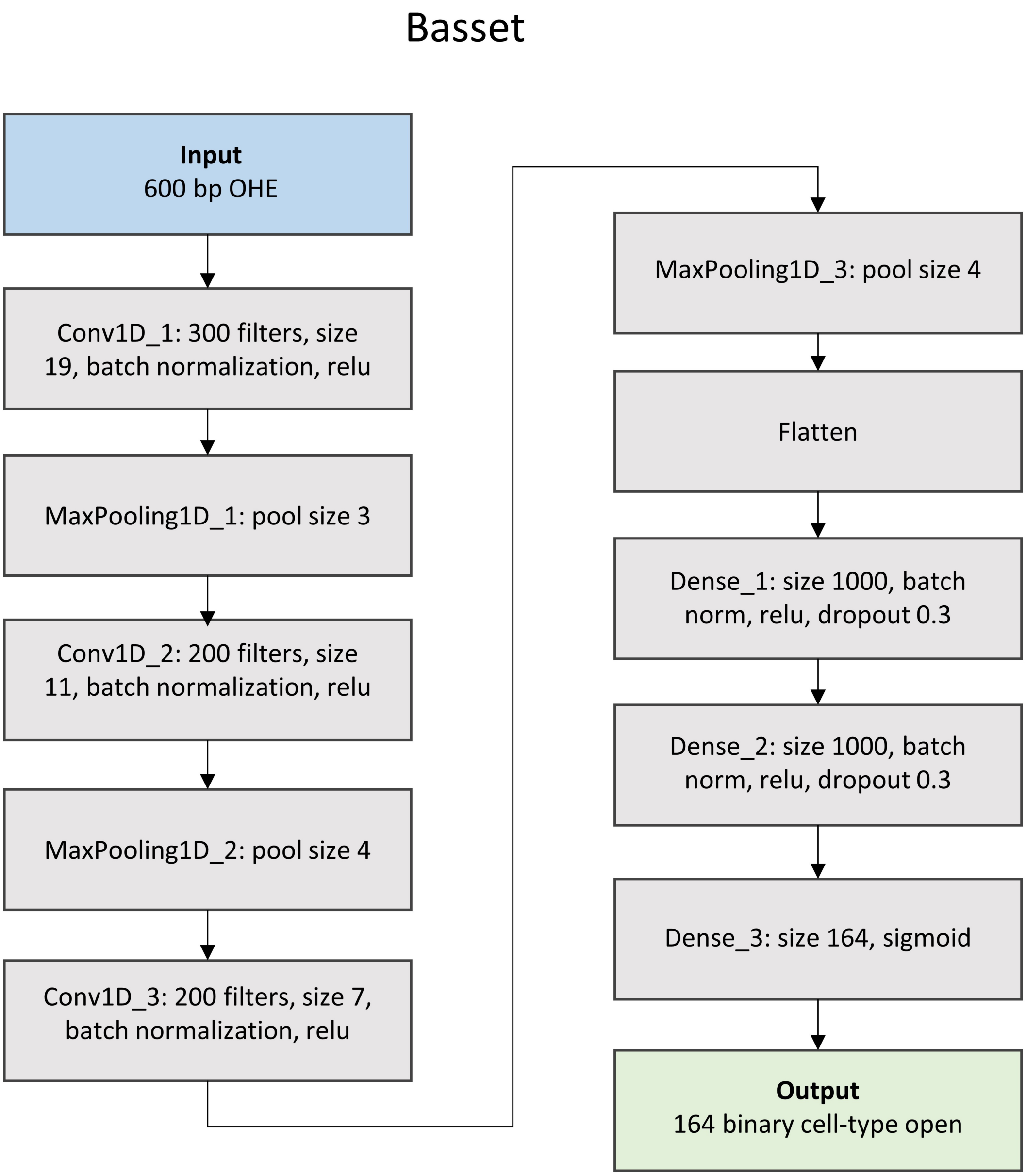

Supplementary Figure 5: **Architecture of the Basset model.** The Basset model (Kelley et al., 2016) was designed to predict the accessibility (binary) in 164 cell-types for a one-hot encoded (OHE) 600bp sequence. The grey boxes represent layers and contain layer name/type and parameters. The blue boxes represent the input and the green box represents the output.

References

B. P. de Almeida, F. Reiter, M. Pagani, and A. Stark. DeepSTARR predicts enhancer activity from DNA sequence and enables the de novo design of synthetic enhancers. *Nature Genetics*, 54(5):613–624, May 2022. ISSN 1546-1718. doi: 10.1038/s41588-022-01048-5.

D. R. Kelley, J. Snoek, and J. L. Rinn. Basset: learning the regulatory code of the accessible genome with deep convolutional neural networks. *Genome Research*, 26(7):990–999, July 2016. ISSN 1549-5469. doi: 10.1101/gr.200535.115.

G. Novakovsky, O. Fornes, M. Saraswat, S. Mostafavi, and W. W. Wasserman. ExplaiNN: interpretable and transparent neural networks for genomics. *Genome Biology*, 24(1):154, June 2023. ISSN 1474-760X. doi: 10.1186/s13059-023-02985-y.
